# SnS-modified platinum electrode for electrocatalytic hydrazine oxidation: mechanistic insights and electrochemical sensing

**DOI:** 10.1039/d6ra06914b

**Published:** 2026-11-06

**Authors:** Mohammad Imran Hossain, S. M. Maksudur Rahman Shanto, Md. Tarikul Islam, Jahir Ahmed, M. Faisal, Jari S. Algethami, Farid A. Harraz, Md Mahmudul Hasan, Md Musfiqur Rahman, Mohammad A. Hasnat

**Affiliations:** a Electrochemistry & Catalysis Research Laboratory (ECRL), Department of Chemistry, School of Physical Sciences, Shahjalal University of Science and Technology Sylhet-3114 Bangladesh imran-che@sust.edu shanto7818@gmail.com tarikulislam038@gmail.com mah-che@sust.edu; b Advanced Materials and Nano-Research Centre (AMNRC), Najran University Najran 11001 Saudi Arabia ursjahir@gmail.com mdfaisalahsan@gmail.com jsalgethami@nu.edu.sa faharraz@nu.edu.sa; c Department of Chemistry, Faculty of Science and Arts, Najran University Najran 11001 Saudi Arabia; d Research Organization for Nano and Life Innovation, Waseda University Tokyo 162-0041 Japan hasan@aoni.waseda.jp; e Department of Mechanical Engineering, Iowa State University Ames Iowa 50011 USA md.musfiqur.rahman@maine.edu

## Abstract

Hydrazine (HZ) is a highly toxic and carcinogenic chemical widely used in industrial, agricultural, and pharmaceutical applications, making its rapid and reliable determination essential for environmental monitoring and public health protection. In this work, a Pt/SnS-modified electrode was developed as an electrochemical sensing platform for sensitive HZ detection. Tin sulfide (SnS) particles were synthesized *via* a hydrothermal method and immobilized onto a Pt surface using a thin PTFE binder layer. The morphology and chemical composition of the modified electrode were characterized by SEM, EDX, Raman spectroscopy, XPS, and XRD confirming the successful formation of the SnS catalyst. Electrochemical investigations revealed that the Pt/SnS electrode exhibited enhanced electrocatalytic activity toward HZ oxidation in alkaline medium compared with bare Pt, producing more than a twofold increase in oxidation current and improved HZ adsorption. Chronoamperometry and convolution potential sweep voltammetry indicated an overall four-electron oxidation process to nitrogen, while kinetic analysis suggested an irreversible multistep oxidation mechanism with an apparent anodic electron-transfer coefficient (*β*) of 0.30–0.32. Differential pulse voltammetry enabled HZ determination over two linear concentration ranges of 5–35 µM and 50–3500 µM, with a limit of detection of 2.68 µM and a limit of quantification of 8.13 µM. The sensor further exhibited good operational stability, reproducibility, selectivity against common interfering species, and satisfactory recoveries of 81.0–99.3% for simulated industrial water samples. These results demonstrate that the Pt/SnS-modified electrode is a prospective electrochemical sensing platform for HZ detection, while the mechanistic electrochemical studies provide fundamental insight into its potent electrocatalytic performance.

## Abstract 2

An α-SnS-modified Pt electrode enhanced hydrazine oxidation by >2-fold. DPV achieved a 2.68 µM detection limit across two linear ranges (5–35 and 50–3500 µM), with 92.1% current retention after 50 cycles.

## Graphical abstract

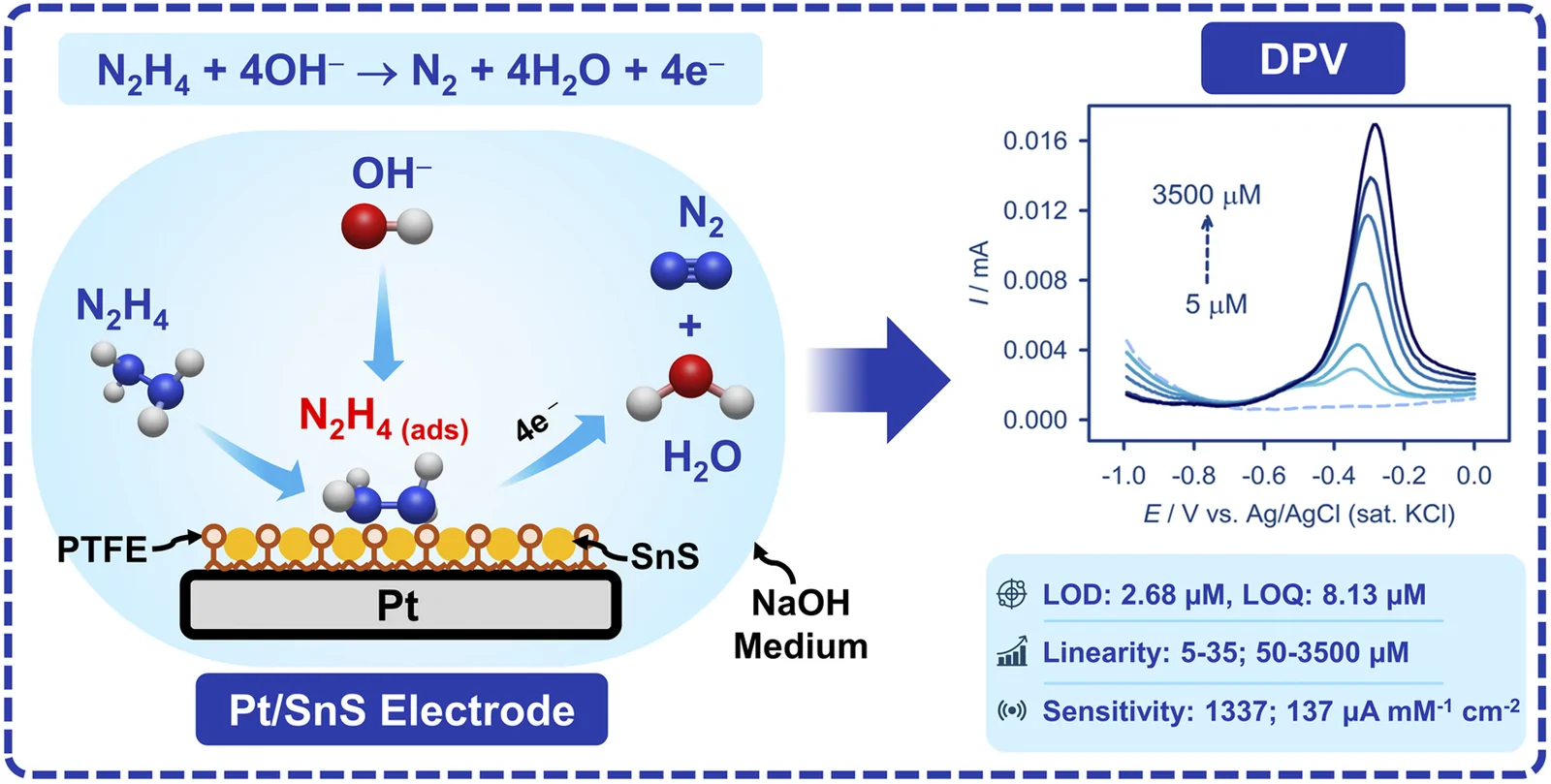

## 1. Introduction

Hydrazine (N_2_H_4_) is an inorganic compound, widely used in chemical industries, pharmaceuticals, corrosion inhibitors, antioxidants, catalysts, polymer manufacturing, emulsifiers, pesticides, insecticides, herbicides, dyestuffs, and explosives.^1–4^ Owing to its high hydrogen content (12.5 wt%) and carbon-free oxidation products, hydrazine (HZ) has also attracted considerable interest as a promising fuel for alkaline direct HZ fuel cells.^5–8^ Despite these advantages, HZ is highly toxic, carcinogenic, and environmentally hazardous even at low concentrations.^9^ The U.S. Environmental Protection Agency (EPA) classifies HZ as a probable human carcinogen (group B2), while the National Institute for Occupational Safety and Health (NIOSH) considers HZ a potential occupational carcinogen.^10,11^ High-level HZ exposure can affect the central nervous system and may irritate the eyes, nose, and throat; it can also damage the lungs, liver, and kidneys.^12–16^ Consequently, the development of rapid, sensitive, and reliable analytical methods for HZ determination is of considerable importance for environmental monitoring, industrial process control, and public health protection.

Several analytical techniques, including spectrophotometry, chromatography, fluorescence spectroscopy, chemiluminescence, and capillary electrophoresis, have been employed for HZ determination.^17–20^ Although these methods generally provide satisfactory analytical performance, they often require sophisticated instrumentation, laborious sample preparation, skilled operators, and relatively high operational costs. In contrast, electrochemical sensing has emerged as an attractive alternative owing to its simplicity, rapid response, low cost, high sensitivity, portability, and suitability for on-site monitoring.^21^ The analytical performance of electrochemical sensors, however, strongly depends on the electrocatalytic properties of the electrode material.

Platinum (Pt) is one of the most effective electrocatalysts for HZ oxidation because of its excellent electrical conductivity, chemical stability, and intrinsic catalytic activity. Nevertheless, bare Pt electrodes often suffer from surface poisoning by adsorbed intermediates, which restrict their analytical performance.^22^ To overcome these limitations, numerous Pt-based composite electrodes have been developed by incorporating transition metals, metal oxides, sulfides, phosphides, and carbon-based materials to enhance catalytic activity and improve electron-transfer kinetics.^23^

Semiconductor–metal composites have attracted considerable attention for chemical sensing because of their catalytic and structural properties.^24–31^ Among these materials, tin sulfide (SnS) has emerged as a promising semiconductor owing to its low toxicity, earth abundance, chemical stability, layered crystal structure, and favorable electronic properties.^32^ The presence of sulfur atoms introduces highly polarizable surface sites capable of interacting strongly with adsorbed molecules, while tin provides additional catalytic centers for surface reactions.^33^ When integrated with Pt, SnS is expected to modify the electronic structure of the Pt surface through interfacial electronic coupling, thereby optimizing the adsorption strength of HZ and its reaction intermediates.^34^ Such synergistic interactions can facilitate charge transfer, increase the density of electrochemically active sites, and promote the oxidation kinetics of HZ.^33,34^ Furthermore, a thin polytetrafluoroethylene (PTFE) binder offers excellent chemical resistance and mechanical stability, enabling firm immobilization of SnS particles on the Pt surface without significantly hindering electrolyte accessibility or long-term electrode stability.^35^

Although several Pt-based composite electrodes have been reported for HZ oxidation, studies employing Pt/SnS heterostructures remain scarce, and the fundamental role of SnS in promoting HZ electrooxidation has not been adequately elucidated. In particular, mechanistic investigations correlating catalytic enhancement with electron-transfer kinetics and analytical sensing performance are limited. Establishing such relationships is essential for the rational design of efficient electrochemical HZ sensors.

Recent developments in chemical sensing highlight the importance of integrating appropriate sensing materials with effective transduction strategies and reliable analytical validation. For example, biogenically synthesized CuO- and Fe_3_O_4_-based electrochemical biosensors have demonstrated the relevance of nanomaterial design, selectivity, and matrix testing in pesticide detection.^36,37^ Beyond conventional electrochemical configurations, advances in field-effect-transistor (FET) sensors and complementary metal-oxide-semiconductor (CMOS) integration offer potential routes toward miniaturized and portable sensing systems.^38^ Recent work on Ti_3_C_2_T_*x*_/CuO-based extended-gate FET sensors has further highlighted the influence of charge-carrier transport in the electronic transducer on sensing performance.^39^ Although these platforms employ different analytes and sensing architectures, they illustrate the broader shift toward sensor designs that consider material properties, interfacial interactions, electronic readout, and practical validation together.

In this work, a Pt/SnS-modified electrode was fabricated by immobilizing hydrothermally synthesized SnS particles onto a Pt substrate using a PTFE binder. The morphology, composition, and chemical states of the modified electrode were characterized by SEM, EDX, Raman spectroscopy, and X-ray photoelectron spectroscopy. The electrocatalytic behavior toward HZ oxidation was systematically investigated using cyclic voltammetry, chronoamperometry, electrochemical impedance spectroscopy, differential pulse voltammetry, and convolution potential sweep voltammetry. Particular emphasis was placed on evaluating the electrode as an electrochemical sensor for HZ through determination of its sensitivity, selectivity, detection limit, and practical applicability in simulated industrial water samples, while mechanistic electrochemical analyses were performed to elucidate the origin of its enhanced sensing performance.

## 2. Experimental

### 2.1. Materials and instrumentation

Hydrazine hydrate (N_2_H_4_·H_2_O), sodium hydroxide (NaOH), sulfuric acid (H_2_SO_4_), and polytetrafluoroethylene (PTFE) solution were obtained from Sigma-Aldrich. Analytical-grade hydrazine hydrate was used to prepare solutions in deionized (DI) water with a resistivity of approximately 18 MΩ·cm. Electrochemical measurements were performed using an Autolab potentiostat (PGSTST 128N, The Netherlands) and a WaveDriver 10 instrument (Pine Research Instrumentation, USA) in a conventional three-electrode configuration. A Pt electrode with a geometric surface area of 0.0314 cm^2^ and a Teflon-protected body was used as the working electrode. A Pt wire and an Ag/AgCl reference electrode containing saturated KCl served as the counter and reference electrodes, respectively. All potentials are reported *versus* Ag/AgCl (sat. KCl).

### 2.2. Synthesis of SnS

SnS was synthesized by dissolving 5 mmol of SnCl_2_·2H_2_O in 15 mL of DI water under magnetic stirring until a homogeneous solution was obtained. Thiourea (4.5 mmol) was then added, and the mixture was stirred for an additional 30 min. The resulting solution was transferred to a 100 mL Teflon-lined autoclave and heated at 200 °C for 24 h. After the autoclave had cooled naturally to room temperature, the resulting precipitate was collected by centrifugation at 6000 rpm, washed with absolute ethanol and DI water using a vortex mixer, and dried at 60 °C for 2 h. The dried product was subsequently used to modify the Pt electrode.

### 2.3. Fabrication of the Pt/SnS electrode

The Pt electrode was mechanically polished on a lapping pad using alumina powder with a particle size of 0.3 µm in a figure-eight motion until a clean surface was obtained. The polished electrode was then sonicated for 20 min at 25 °C in a cleaning medium containing 0.5 M H_2_SO_4_ and acetone to remove surface contaminants. Electrochemical cleaning was subsequently performed by cycling the electrode in 0.1 M H_2_SO_4_ between 0.2 and +1.5 V *versus* Ag/AgCl (sat. KCl) at a scan rate of 0.1 V s^−1^ until successive cyclic voltammograms became stable and reproducible.

To prepare the catalytic ink, 1 mg of the synthesized SnS catalyst was mixed with a 5 wt% PTFE solution diluted with ethanol. A 6 µL aliquot of the resulting ink was drop-cast onto the cleaned Pt electrode (geometric surface area = 0.0314 cm^2^) using a micropipette. The modified electrode was then heated in a vacuum oven at 80 °C for 10 min. PTFE served as a binder to immobilize the SnS particles on the Pt surface.

### 2.4. Electrochemical measurements

Electrochemical measurements were performed in a conventional three-electrode cell using the Pt/SnS electrode as the working electrode, a Pt wire as the counter electrode, and Ag/AgCl (sat. KCl) as the reference electrode. Linear polarization curves were recorded in 0.1 M NaOH under N_2_-saturated conditions, both in the absence and presence of hydrazine (HZ). Electrochemical impedance spectroscopy (EIS) measurements were conducted in 0.1 M NaOH containing 2 mM HZ at an excitation potential of −0.35 V. The effect of HZ concentration was investigated by cyclic voltammetry (CV) following successive additions of HZ to 0.1 M NaOH. The influence of scan rate on mass transport was examined by CV in 10 mL of 0.1 M NaOH containing 1.5 mM HZ, using scan rates ranging from 0.01 to 0.4 V s^−1^. N_2_ gas was used to maintain an inert atmosphere during the electrochemical measurements. Data analysis and graphical presentation were carried out using SigmaPlot v.10 and OriginPro 2025b.

### 2.5. Materials characterization

The surface morphology and elemental composition of the synthesized material were examined using scanning electron microscopy coupled with energy-dispersive X-ray spectroscopy (SEM–EDX; SmartEDX, ZEISS, Germany). Raman spectra were recorded using an Ava Raman spectrometer equipped with a 785 nm laser (Avantes, The Netherlands). Powder X-ray diffraction (PXRD) patterns were recorded using a Rigaku SmartLab diffractometer with Cu Kα radiation (*λ* = 1.5406 Å). Le Bail fitting of the PXRD pattern was performed using TOPAS 3 (Bruker AXS) to evaluate the crystalline phase and refine the lattice parameters. The chemical states of the synthesized material were investigated by X-ray photoelectron spectroscopy (XPS) using a Kratos Axis Ultra spectrometer equipped with a delay-line detector (DLD) and an Al Kα radiation source (1486.6 eV). Open-circuit potential (OCP) and electrochemical impedance spectroscopy (EIS) measurements were performed using an Autolab potentiostat (PGSTST 128N, The Netherlands).

## 3. Results and discussions

### 3.1. Structural and chemical characterization of the SnS catalyst

The morphology and composition of the synthesized SnS catalyst were characterized by SEM, EDX, and Raman spectroscopy. Representative SEM micrographs recorded at magnifications of 3000× and 100 00× (Fig. 1A and B) reveal a relatively uniform distribution of SnS particles with micrometer-sized features over the sample surface. The EDX spectrum (Fig. 1C) confirms Sn and S as the principal constituent elements, with atomic percentages of 68.1% and 31.9%, respectively. Although the measured Sn/S ratio deviates from the ideal 1 : 1 stoichiometry of SnS, the elemental analysis confirms the presence of Sn and S as the major constituents. Further structural confirmation was obtained from Raman spectroscopy. As shown in Fig. 1D, a characteristic Raman band centered at 320 cm^−1^ is assigned to the *A*_g_ vibrational mode of SnS, which is in good agreement with previously reported values for crystalline SnS, thereby confirming the successful synthesis of the sulfide phase.^40–42^

**Fig. 1:**
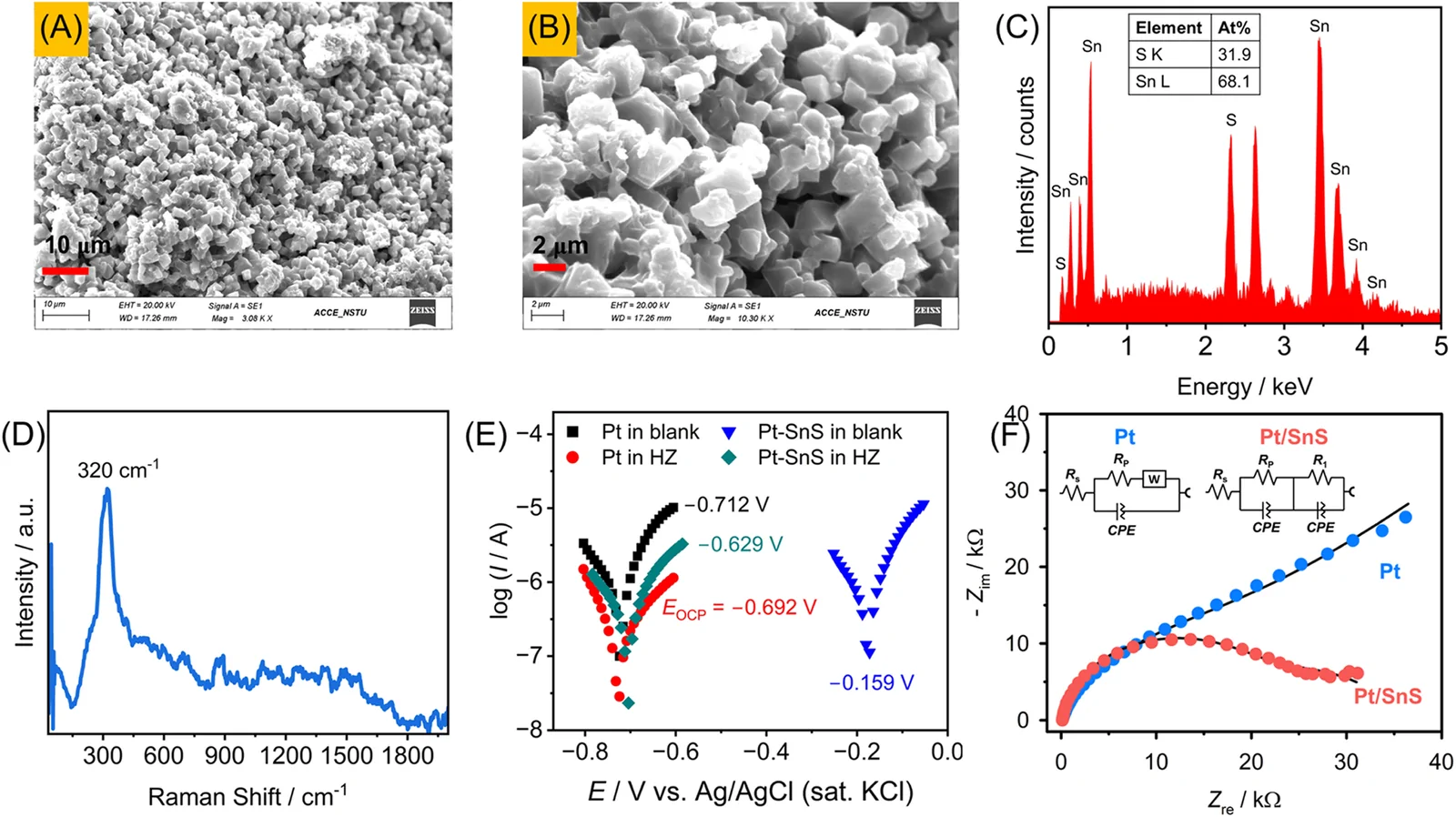
SEM images of SnS catalyst at (A) 3000- and (B) 10 000-times magnification. (C) EDX elemental spectra of the synthesized SnS catalyst. (D) Raman spectra of SnS microparticles. (E) Linear polarization curves for determining open circuit potential of Pt and Pt/SnS electrode surfaces in presence and absence of HZ. (F) Nyquist plots showing experimental data points and corresponding equivalent-circuit fitting curves (solid lines) for bare Pt and SnS-modified Pt (Pt/SnS) electrodes recorded at −0.35 V *vs.* Ag/AgCl (sat. KCl) in 0.1 M NaOH containing 2 mM hydrazine. The equivalent circuits used for fitting are shown in the inset.

The electrochemical characteristics of the catalyst surface were first investigated by linear polarization and electrochemical impedance spectroscopy. To probe the interaction between HZ molecules and the electrode surface, linear polarization curves were recorded in 0.1 M NaOH in both the absence and presence of HZ. Fig. 1E compares the polarization behavior of pristine Pt and Pt/SnS electrodes under identical experimental conditions. The open-circuit potential (OCP) values revealed the difference in responses for the pristine Pt and Pt/SnS electrodes following the addition of HZ. For pristine Pt, the OCP shifted from −0.712 V in blank 0.1 M NaOH to −0.692 V after HZ addition, corresponding to a positive shift of 20 mV. In contrast, the OCP of the Pt/SnS electrode shifted from −0.159 V to −0.629 V, corresponding to a negative shift of approximately 470 mV. This substantially larger potential displacement indicates that the open-circuit electrochemical equilibrium of the modified electrode was more strongly perturbed by the introduction of HZ under the conditions investigated. The observed potential shift is interpreted as evidence of a pronounced interfacial response to HZ addition on the SnS modified Pt in comparison to the bare Pt electrode.

Electrochemical impedance spectroscopy (EIS) was employed to investigate the interfacial impedance characteristics of bare Pt and Pt/SnS electrodes in 0.1 M NaOH containing 2 mM HZ. The experimental Nyquist plots (shown in Fig. 1F) were fitted using the equivalent circuits presented in the inset of Fig. 1F, and the corresponding fitting parameters, estimated errors, and goodness-of-fit statistics are summarized in Table S1.

The impedance spectrum of bare Pt was fitted using a circuit comprising the solution resistance (*R*_s_), a constant-phase element (CPE) in parallel with a branch containing the resistance (*R*_p_) and Warburg diffusion element (W). The fitted values of (*R*_s_) and (*R*_p_) were 116.42 Ω and 20.760 kΩ, respectively, with a *χ*^2^ value of 0.063293. In comparison, the Pt/SnS spectrum required a more complex equivalent circuit containing two resistive-CPE elements connected in series after the solution resistance. The fitted parameters were *R*_s_ = 123.68 Ω, *R*_p_ = 19.889 kΩ, and *R*_1_ = 15.550 kΩ, with a *χ*^2^ value of 0.063193.

Notably, the principal fitted resistance parameter (*R*_p_) decreased by approximately 4.2% following SnS modification, from 20.760 to 19.889 kΩ. Although this modest decrease cannot be interpreted as a definitive reduction in the overall charge-transfer resistance because the two electrodes were fitted using different circuit topologies, it indicates that the principal resistive contribution (*R*_p_) is comparable for the two electrodes and is slightly lower for Pt/SnS. The additional *R*_1_ ‖ CPE element in the Pt/SnS model indicates an additional relaxation contribution associated with the modified electrode. This contribution may be related to the SnS/PTFE-containing surface layer or an additional interfacial process; however, its precise physical origin cannot be uniquely established from the present impedance data.

The chemical composition and oxidation states of the synthesized SnS catalyst were further investigated by X-ray photoelectron spectroscopy (XPS). The survey spectrum (Fig. 2A) confirms the presence of Sn, S, and O as the major constituent elements of the catalyst. The high-resolution Sn 3d spectrum (Fig. 2B) exhibits two characteristic peaks centered at 487.9 eV and 496.3 eV, corresponding to the Sn 3d_5/2_ and Sn 3d_3/2_ spin–orbit components, respectively, which are characteristic of Sn species in SnS. These two peaks are separated by approximately 8.4 eV, in good agreement with previously reported values for SnS.^43^ The deconvoluted S 2p spectrum (Fig. 2C) displays a well-defined doublet with binding energies of 163.6 eV and 164.7 eV, assigned to the S 2p_3/2_ and S 2p_1/2_ states of sulfide (S^2−^) in SnS, respectively.^44^ In addition, a distinct peak at 169.7 eV is attributed to oxidized sulfur species, which likely originated from partial surface oxidation of the catalyst upon exposure to air.

**Fig. 2:**
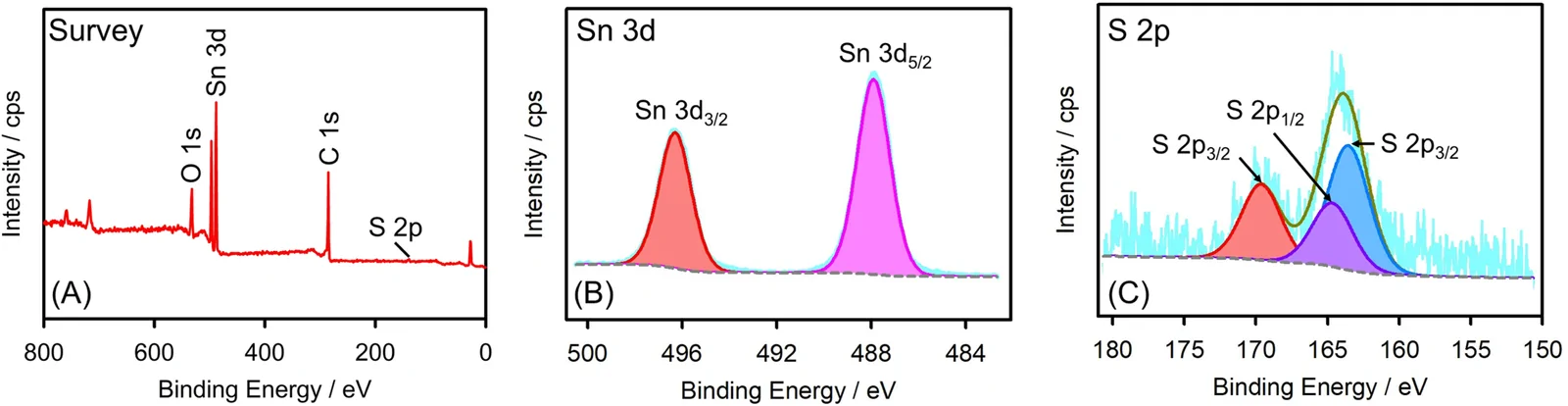
(A) XPS survey spectrum for SnS catalyst. Deconvoluted XPS spectra of (B) Sn 3d and (C) S 2p regions for SnS particles.

To further examine possible surface transformation during alkaline electrochemical operation, the SnS catalyst was characterized by XPS after completion of the electrochemical sensing experiments. The O 1s spectrum (given in Fig. S1) exhibits two components at measured binding energies of 531.65 and 532.51 eV, corresponding to corrected binding energies of 530.59 and 531.45 eV, respectively. The lower-binding-energy component at 530.59 eV is assigned to surface-bound hydroxyl groups (Sn–OH), oxygen-deficient lattice coordination sites, or surface-terminating oxygen species associated with the SnS surface.^45,46^ The component at 531.45 eV is attributed to physisorbed/chemisorbed ambient water (H_2_O_ads_).^45,47^ These features indicate the presence of oxygen-containing surface species after exposure to the alkaline electrolyte; however, the O 1s spectrum does not show a distinct component that can be unambiguously assigned to a separate SnO phase. Therefore, the post-electrochemical XPS results do not provide direct evidence for substantial conversion of SnS into SnO.

The crystalline phase of the synthesized tin sulfide material was investigated by X-ray diffraction (XRD). As shown in Fig. S2(A), the experimental diffraction pattern was compared with simulated patterns of α-SnS (*Pnma*), β-SnS (*Cmcm*), π-SnS (*P*312), SnS_2_ (*P*3̄*m*1), and Sn_2_S_3_ (*Pnma*) to evaluate the phase assignment and identify possible secondary phases. The experimental reflections show good agreement with the simulated pattern of orthorhombic α-SnS, supporting its assignment as the predominant crystalline phase in the synthesized material. No detectable characteristic reflections attributable to β-SnS, π-SnS, SnS_2_, or Sn_2_S_3_ were observed in the experimental pattern, indicating that these phases were not detected within the sensitivity of the XRD measurement. Furthermore, Le Bail fitting of the room-temperature XRD pattern yielded an *R*_wp_ value of 0.2108 and a goodness-of-fit value of 1.11 (Fig. S2(B)). The refined lattice parameters were *a* = 11.1850(6) Å, *b* = 3.9849(5) Å, and *c* = 4.3145(3) Å, consistent with the orthorhombic α-SnS structure. Overall, the agreement between the experimental diffraction pattern, the simulated α-SnS pattern, and the refined lattice parameters provides additional crystallographic evidence supporting the assignment of the synthesized material to α-SnS.

### 3.2. Electrocatalytic activity toward HZ oxidation

The electrocatalytic activity of the Pt/SnS electrode toward HZ oxidation was evaluated by cyclic voltammetry (CV) in 0.1 M NaOH. As shown in Fig. 3A, the Pt/SnS electrode recorded in the absence of HZ (dashed line) exhibits only a negligible background current over the investigated potential window, indicating that the modified electrode does not undergo significant faradaic processes in the supporting electrolyte.

**Fig. 3:**
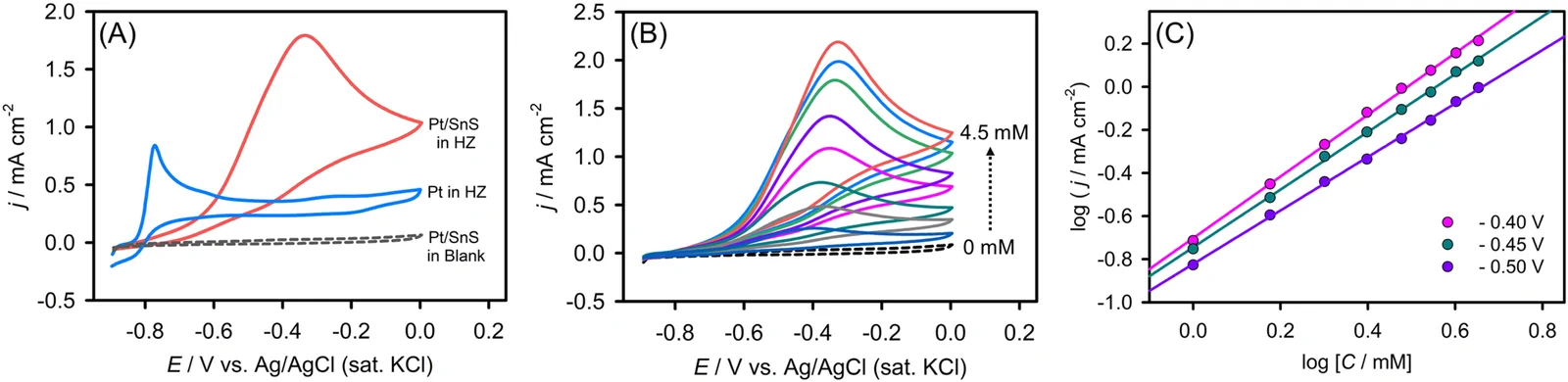
(A) Cyclic voltammograms (CVs) of 3 mM HZ solution recorded in 0.1 M NaOH medium at a scan rate of 0.05 V s^−1^ using bare Pt and SnS-modified Pt electrode. (B) Concentration-dependent CVs for the oxidation of HZ in 0.1 M NaOH at modified Pt/SnS electrode with a scan rate of 0.05 V s^−1^. (C) The correlation between logarithm of current density (*j*) and logarithm of HZ concentration at three specific potentials (−0.40 V, −0.45 V, and −0.50 V).

Upon the addition of HZ, the bare Pt electrode exhibits an oxidation wave with an onset potential of approximately −0.83 V (*vs.* Ag/AgCl (sat. KCl)), followed by a relatively sharp anodic peak centered at −0.77 V. Although HZ oxidation is initiated at a comparatively lower overpotential on the pristine Pt surface, the oxidation current reaches a maximum of only 0.81 mA cm^−2^ before decreasing rapidly at more positive potentials. Such behavior suggests that the catalytic activity of bare Pt is not sustained over a broad potential range. In contrast, the Pt/SnS electrode displays a substantially different voltammetric response. The oxidation process begins at approximately −0.65 V, followed by a broad anodic wave centered near −0.34 V. Despite the slightly more positive onset potential, the modified electrode produces a notably higher peak current density of approximately 1.8 mA cm^−2^, corresponding to more than a twofold enhancement compared with the bare Pt electrode. Moreover, the broad oxidation wave extends over a wider potential region without the abrupt current decay observed for pristine Pt, indicating that the catalytic surface remains active throughout the oxidation process.

Here, the enhanced oxidation current and sustained catalytic response demonstrate that immobilization of SnS on the Pt surface considerably improves the electrocatalytic performance toward HZ oxidation. The improved activity is consistent with the formation of additional catalytically active interfacial sites and more favorable adsorption and activation of HZ on the Pt/SnS interface. These observations agree well with the pronounced OCP shift and the kinetic analyses presented in the subsequent sections. The larger oxidation current together with the negligible background current recorded in the blank electrolyte provides a higher signal-to-background ratio, which makes the Pt/SnS electrode a viable candidate for conducting sensitive electrochemical HZ detection. Moreover, to establish the most suitable candidacy as a substrate for SnS modification, Pt was compared with other commonly used electrode materials in identical experimental conditions. As shown in Fig. S3, Au/SnS exhibits an HZ oxidation peak at −0.252 V with a low peak current density of only 0.286 mA cm^−2^, while GC/SnS produces no well-defined oxidation peak. The superior electrochemical response of Pt confirms its suitability as the substrate for SnS modification.

Fig. 3B presents the concentration-dependent cyclic voltammograms recorded for the electrocatalytic oxidation of HZ at the Pt/SnS electrode. As the HZ concentration increased, the oxidation peak current increased monotonically, demonstrating good electrocatalytic response of the modified electrode toward HZ. Simultaneously, the oxidation peak shifted slightly toward more positive potentials with increasing HZ concentration, suggesting that a progressively higher overpotential is required to sustain the oxidation process at elevated analyte concentrations. This behavior can be attributed to increased surface coverage of adsorbed species and the accompanying concentration overpotential requiring for HZ oxidation. The difference between the peak potential and the half-peak potential (Δ*E*_p_) remained within the range of 0.040–0.048 V, which is characteristic of an irreversible electrode process.^48^ Moreover, the relatively small Δ*E*_p_ values indicate rapid heterogeneous electron-transfer kinetics and efficient charge transport at the Pt/SnS interface despite the irreversible nature of the oxidation reaction. To gain further insight into the reaction kinetics, the kinetic order (*γ*) with respect to HZ concentration was determined from the concentration-dependent voltammograms using the following eqn (1).

1 log *j* = log *k* + *γ* log[HZ]

where, *j* is the oxidation current density, *k* is the apparent kinetic rate constant, and *γ* is the kinetic order of the reaction. Fig. 3C shows the plots of log *j versus* log[HZ] obtained at applied potentials of −0.40, −0.45, and −0.50 V. The corresponding kinetic orders were determined to be 1.03, 1.05, and 1.07, respectively. The values close to unity demonstrate that the electrocatalytic oxidation of HZ follows first-order reaction kinetics with respect to HZ concentration over the investigated potential range. The observed first-order behavior indicates that the oxidation rate is directly proportional to the HZ concentration in solution. Such linear concentration dependence is particularly advantageous for electrochemical sensing because the oxidation current changes proportionally with analyte concentration, enabling straightforward calibration.

At this point, it was important to evaluate the number of electron transfer involved to predict the oxidized product generated due to HZ oxidation reaction at Pt/SnS electrode. For this purpose, chronoamperograms (CAs) of 2 mM HZ in 0.1 M NaOH solution were recorded systematically by stepping the potential by 50 mV from −0.6 to −0.2 V. It was observed that the data received at potentials less negative than −0.6 V matched well with the well-known Cottrell equation shown as eqn (2).^49^2
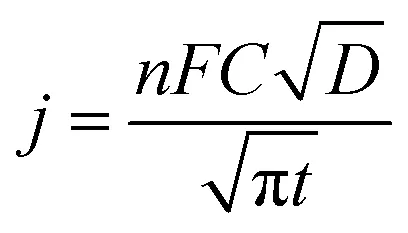
where, *j* = current density (mA cm^−2^), *n* = electron number, *C* = concentration of analyte (mol cm^−3^), *D* = diffusion coefficient expressed as cm^2^ s^−1^ which is 1.4 × 10^−5^ in the present case),^50^*t* = time (s).

The apparent number of electrons transferred during hydrazine oxidation was estimated from the slope of the current density *versus t*^−1/2^ plot (given as inset of Fig. 4) obtained from the chronoamperometric data at −0.45 V. The calculated electron number was approximately 3.71, which is close to the theoretical value of four electrons for the overall oxidation of hydrazine to nitrogen. Assuming nitrogen as the final oxidation product, the corresponding net reaction in alkaline medium can be proposed as per following eqn (3).^51^

3 N_2_H_4_ + 4OH^−^ → N_2_ + 4H_2_O + 4e^−^

**Fig. 4:**
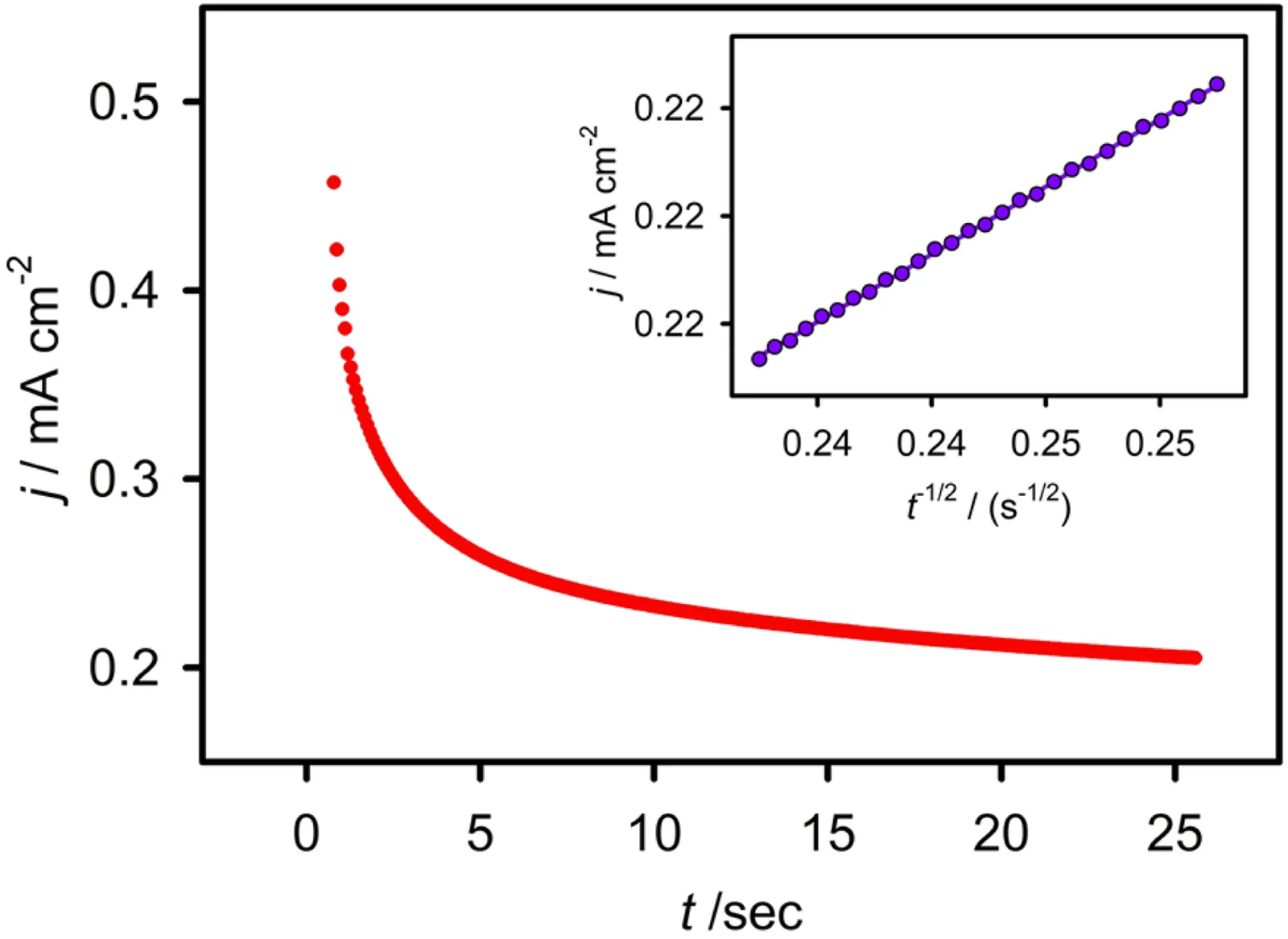
Chronoamperogram (CA) of 2.0 mM HZ at Pt/SnS electrode recorded at −0.45 V. Inset displays relationship between diffusional current density and inverse of square root of time.

### 3.3. Electron-transfer kinetics and reaction mechanism

The effect of scan rate on HZ oxidation provides important insights into the underlying electrochemical mechanism. Fig. 5A presents the CVs of 1.5 mM HZ in 0.1 M NaOH recorded over a scan rate range of 0.01–0.4 V s^−1^. As the scan rate increases, the peak current (*j*_p_) increases systematically, accompanied by a noticeable positive shift in the peak potential (*E*_p_).

**Fig. 5:**
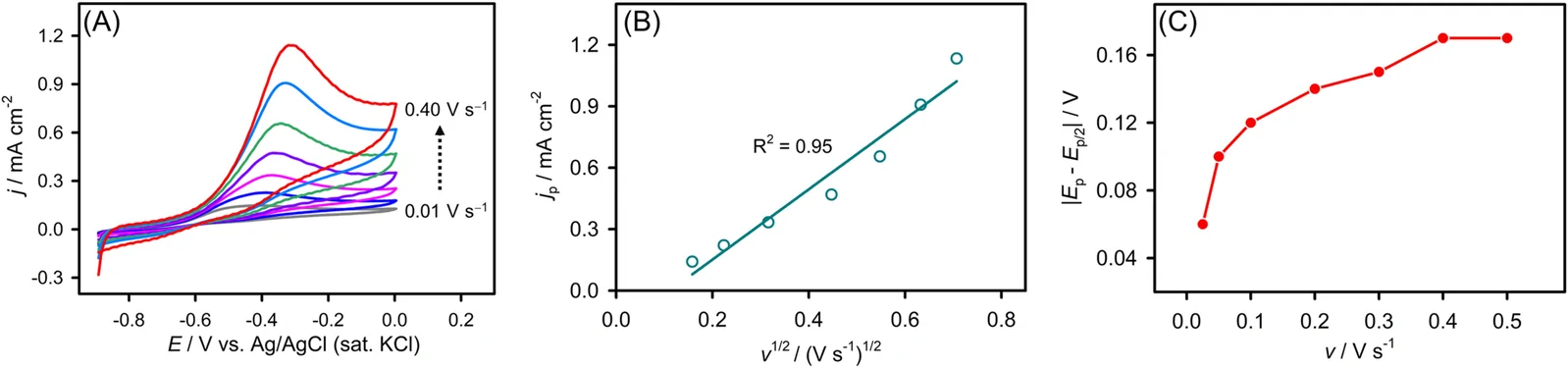
(A) Scan rate-dependent CVs of 1.5 mM HZ in 0.1 M NaOH solution recorded using Pt/SnS electrode. (B) Correlation between peak current density (*j*_p_) and square root of scan rate (*v*^1/2^). (C) Variation of |*E*_p_ −*E*_p/2_*|* as a function of scan rate (*v*). Here *E*_p_ is the peak potential and *E*_p/2_ is the half-peak potential.

A linear relationship between *j*_p_ and the square root of the scan rate (*ν*^1/2^) is observed (*R*^2^ = 0.95), as shown in Fig. 5B, suggesting that the overall process is diffusion-controlled. However, further kinetic analysis reveals deviations from ideal behavior. Specifically, the difference between the peak potential and half-peak potential (|*E*_p_ −*E*_p/2_*|*) increases progressively with scan rate, as illustrated in Fig. 5C, due to the continuous anodic shift of *E*_p_ at higher scan rates.

For the HZ oxidation reaction, the magnitude of (|*E*_p_ −*E*_p/2_*|*) increases considerably from 60 mV to 170 mV with increasing scan rate. Such pronounced variation is characteristic of an irreversible electrochemical process.^52^ Moreover, this deviation indicates that the reaction kinetics do not strictly follow classical Butler–Volmer equation behavior. Instead, it suggests the involvement of complex, multi-step electron transfer processes.^53,54^

In such case, convolution potential sweep is the most convenient tool to study the electron transfer process where all the data points are used from the original CV to carry out analysis.^55,56^ Furthermore, it is also used to study the kinetic and thermodynamic behavior of electron transfer processes.^48^ The convolution current *I*(*t*) was calculated from the voltammetric response using the integral transformation as per following eqn (4).^57^4
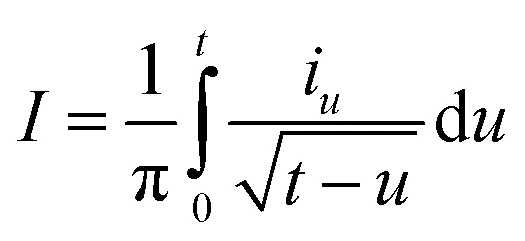
where, *i*(*u*) represents the experimentally obtained current at time *u*. The transformation of the voltammetric data resulted in a characteristic sigmoidal curve as shown in Fig. 6A (dotted line), which is a hallmark of diffusion-controlled electrochemical processes. At sufficiently positive potentials, the convolution current reaches a steady-state plateau corresponding to the limiting convolution current (*I*_l_). The limiting convolution current is expressed by the following eqn (5).^58,59^

5 *I*_l_ = *nFAC*√*D*

where *n* is the number of electrons transferred, *F* is the Faraday constant, *A* is the electrode area, *C* is the bulk concentration of HZ, and *D* is its diffusion coefficient. The limiting current (*I*_l_) was found nearly independent of scan rate (see Fig. 6B) and exhibited an average value of approximately 4.66 × 10^−4^ A. This behavior is advantageous for the accurate determination of *n*, provided that the background capacitive current is properly corrected. By considering a diffusion coefficient value of 1.4 × 10^−5^ cm^2^ s^−1^ (ref. 50) and an electrode surface area of 0.0314 cm^2^, the number of electrons transferred was calculated to lie in the range of 3.71–3.83. These values are close to 4, indicating a four-electron (4e^−^) transfer process for HZ oxidation, which is consistent with previously reported results.

**Fig. 6:**
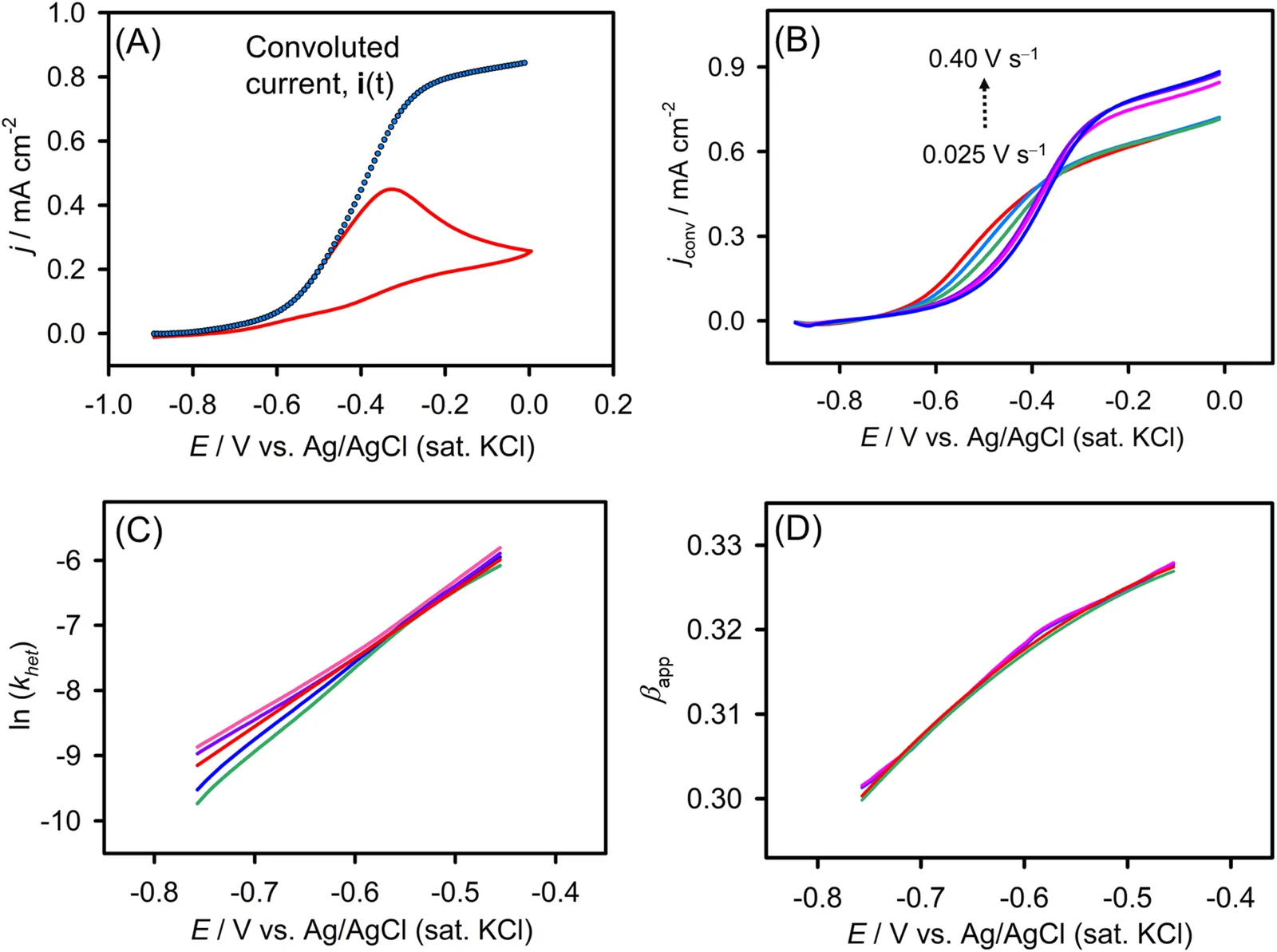
(A) CV of 1.5 mM HZ in 0.1 M NaOH at scan rate of 0.01 V s^−1^. Here the dotted line indicates the convoluted current for the forward scan only. (B) Convoluted voltammograms derived from the CVs recorded within the scan rate of 0.01 V s^−1^ to 0.40 V s^−1^. (C) Dependency of ln *k*_het_ on applied potential at different scan rates. (D) Variation of transfer coefficient (*β*) as a function of potential applied during HZ oxidation by Pt/SnS electrode at different scan rates.

The potential-dependent heterogeneous rate constant, *k*(*E*) (also expressed as *k*_het_), for an irreversible process can be evaluated from eqn (6), which is shown in Fig. 6C.6
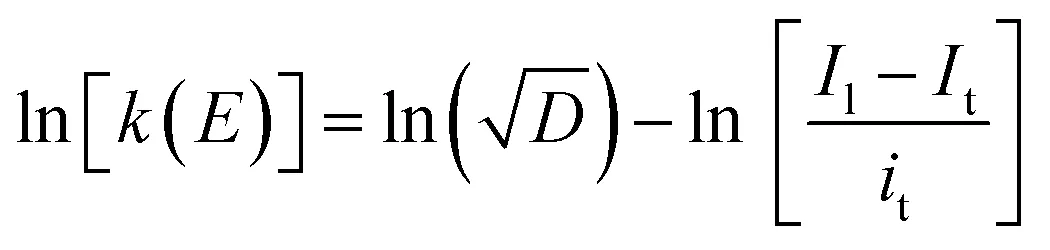
where *I*_l_ is the limiting convolution current (4.66 × 10^−4^ A), *I*_*t*_ is the time-dependent convolution current, and *i*_t_ is the cyclic voltammetric current.^38^ In Fig. 6C, the increase in ln *k*_het_ with applied potential (*E*) indicates that the heterogeneous electron transfer process at the electrode surface is strongly potential-dependent. The apparent transfer coefficient (*β*_app_) was subsequently determined from the slope of the ln *k*_het_*vs. E* plots by linear regression within a narrow potential window, according to eqn (7).^40,41^7
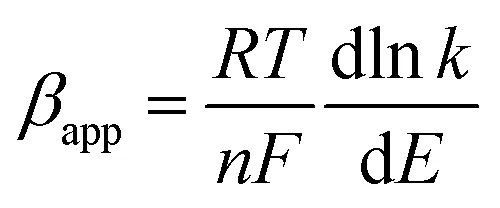


In Fig. 6D, the transfer coefficient (*β*) is observed to vary within a narrow range of 0.30–0.32 over the potential window of −0.75 to −0.45 V at different scan rates. The consistently low value of *β* (<0.5) suggests that the electron transfer process might be highly asymmetric and kinetically sluggish in the forward direction. Such behavior is typically indicative of a rate-determining step (RDS) involving the formation of a reactive intermediate species rather than a direct concerted electron transfer. In particular, a *β* value considerably lower than 0.5 implies that the transition state might be closer to the reactant side, with the involvement of a slow initial electron transfer step that may lead to the generation of a radical intermediate (˙N_2_H_4_).^42,60–62^ This observation indicates a multi-step electron transfer mechanism in which the formation of the intermediate is slower than the subsequent oxidation steps.^63^

HZ oxidation at the Pt/SnS electrode proceeds *via* heterogeneous electron transfer steps coupled with surface adsorption phenomena. As per our previous calculation, the overall oxidation involves a four-electron transfer process. In alkaline media, this reaction is likely preceded by a chemical pre-adsorption step, where HZ molecules accumulate near and partially adsorb onto the electrode surface. The imposed waiting time prior to measurement facilitates this pre-accumulation, enhancing surface coverage and promoting interfacial reactions. Based on these observations, the oxidation mechanism at the Pt/SnS interface can be proposed as a sequence of chemical and electrochemical steps. Initially, HZ interacts with the catalyst surface in the presence of hydroxide ions, leading to partial dehydrogenation and the release of the first electron as shown in eqn (8).

8 *N_2_H_4_ + OH^−^ → *N_2_H_3_ + H_2_O + e^−^

This is followed by a second electron transfer step that might generate a highly reactive radical intermediate (eqn (9)).9
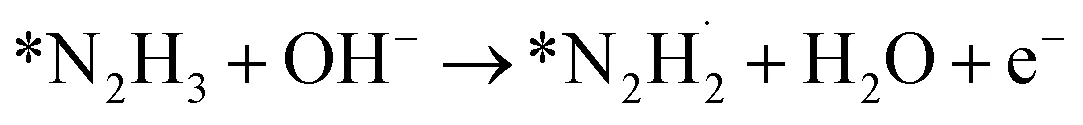


The formation of this radical species is kinetically slow, consistent with the low *β* values obtained from CPSV analysis. Once formed, the radical intermediate undergoes rapid subsequent oxidation steps due to its high reactivity, leading to the formation of molecular nitrogen (eqn (10)).10
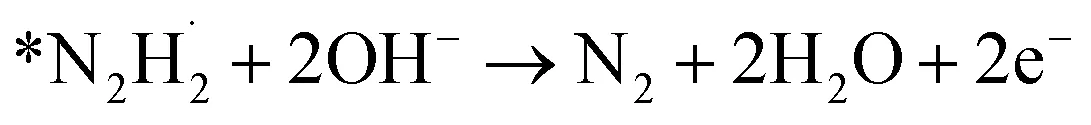


The final steps are expected to be significantly faster, as radical species readily undergo oxidation. Overall, this plausible mechanistic pathway highlights that HZ oxidation at the Pt/SnS electrode is governed by a multi-step kinetic pathway.

### 3.4. Electrochemical sensing performance toward HZ

To evaluate the feasibility of the Pt/SnS electrode as a cost-effective and reliable electrochemical sensor for HZ quantification in aqueous media, differential pulse voltammetry (DPV) measurements were performed at room temperature in 0.1 M NaOH containing various concentrations of HZ (5–3500 µM). The corresponding DPV responses are presented in Fig. 7A. The Pt/SnS-modified electrode exhibited a concentration-dependent increase in oxidation peak current, revealing two distinct linear concentration ranges of 5–35 µM and 50–3500 µM, as shown in Fig. 7B. The calibration curve for the lower concentration range was described by the equation *y* = 4.2 × 10^−5^*x* − 9.9 × 10^−5^, with a correlation coefficient (*R*^2^) of 0.9932. For the higher concentration range, the linear equation was *y* = 4.3 × 10^−6^*x* + 6.4 × 10^−4^, with an (*R*^2^) value of 0.9924. Here, *x* represents the HZ concentration (µM), and *y* represents the oxidation peak current (mA). The corresponding slopes were 4.2 × 10^−5^ and 4.3 × 10^−6^ mA µM^−1^ for the lower and higher concentration ranges, respectively. Based on the geometric electrode area of 0.0314 cm^2^, the corresponding current–density-normalized sensitivities were 1.34 × 10^−3^ and 1.37 × 10^−4^ mA cm^−2^ µM^−1^, respectively. The limit of detection (LOD) was calculated using the slope of the lower-concentration calibration curve and the standard deviation of five blank measurements according to eqn (11) and (12).

11 SD = SE × *N*

12
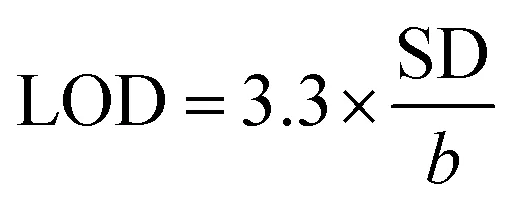
where SD is the standard deviation, SE is the standard error of the blank measurements (1.527 × 10^−8^ A), *N* is the number of blank measurements (5), and *b* is the slope of the lower-concentration calibration curve (4.2 × 10^−8^ A µM^−1^). Based on these equations, the LOD of the Pt/SnS electrode for HZ detection was determined to be 2.68 µM. Similarly, the limit of quantification (LOQ) was calculated using eqn (13).13
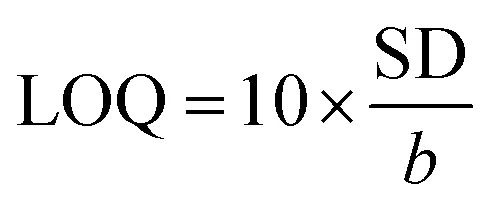


**Fig. 7:**
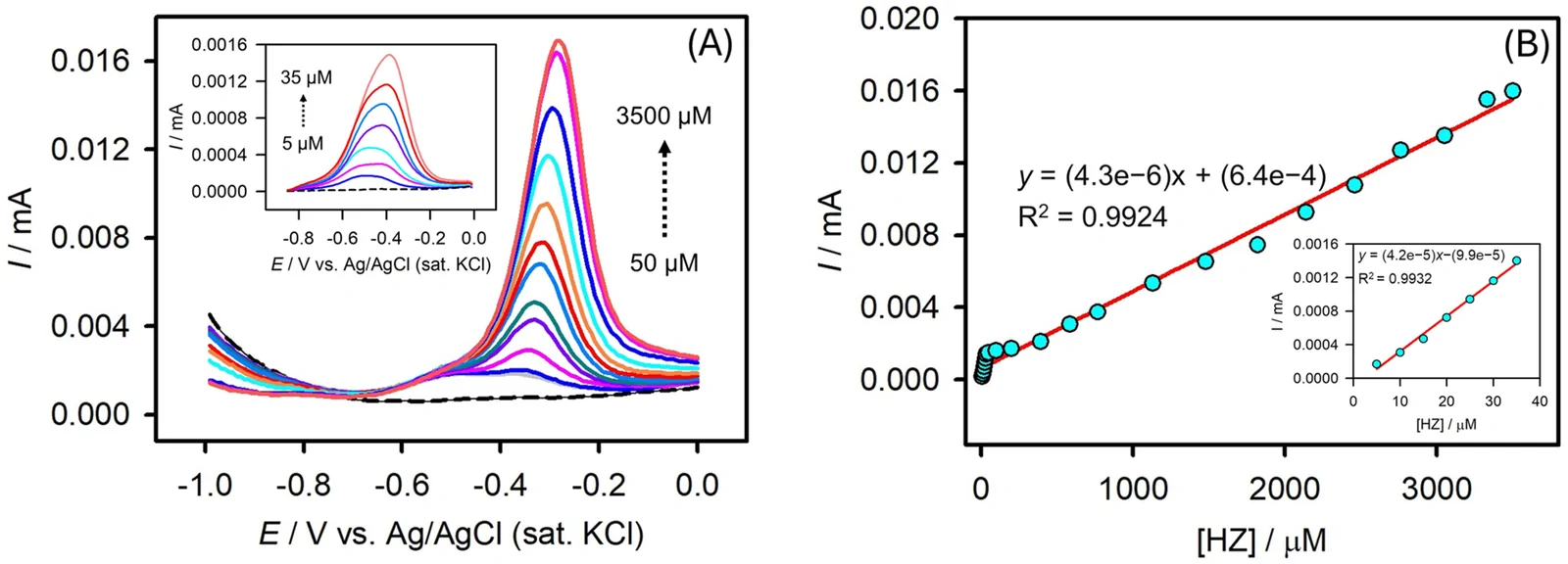
(A) Differential pulse voltammograms (DPVs) of 50 to 3500 µM HZ oxidation response in N2-saturated 0.1 M NaOH recorded using Pt/SnS electrode. The inset shows the detailed DPVs for lower concentration ranging from 5 to 35 µM HZ. (B) Correlation between peak current (*I*_p_) and concentration of HZ. DPV measurements were carried out using a step potential of 8 mV and a pulse amplitude of 25 mV.

The LOQ of the Pt/SnS electrode was calculated to be 8.13 µM, indicating its capability for quantitative HZ determination above this concentration within the lower linear range. The two distinct linear ranges demonstrate the concentration-dependent analytical response of the Pt/SnS electrode over a broad concentration interval. The Pt/SnS electrode exhibits comparable performance to that of previously reported noble metal-based electrodes for HZ detection, as summarized in Table 1.

**Table 1:** Comparison of Pt/SnS with several previously reported noble metal based-electrodes for HZ oxidation

Electrode	Method	Medium	Linearity (µM)	Sensitivity (µA mM^−1^ cm^−2^)	LOD (µM)	Ref.
Pt/HNPG^a^	CA^g^	0.2 M PBS	5–6105	3449.7	1.03	64
Ni-DAP/Au-PtNPs/NFs/GC^b^	CA	0.1 M NaOH	0.2–85	—	0.1	65
GO/CTS/Pt/GCE^c^	CA	0.1 M PBS	20–10000	104.6	3.6	66
Nano-Au/Ti^d^	CV	1 M NaOH	500–4000	1.117	42	67
PdNPs-EDAC/GCE^e^	DPV	0.1 M PBS	5–100	403.7	1.5	68
Pd/BMWCNTs^f^	LSV^h^	0.1 M H_2_SO_4_	56–157	—	10	69
Pt/SnS	DPV	0.1 M NaOH	5–35; 50–3500	1337; 137	2.68	This work

a Highly surface roughened nanoporous gold electrode decorated with Pt nanoparticles.

b Nickel-2,6-diaminopyridine (Ni-DAP) on bimetallic Au–Pt inorganic–organic hybrid nanocomposite modified glassy carbon electrode.

c Graphene oxide/chitosan/platinum-modified glassy carbon electrode.

d Nanoporous gold particles modified titanium electrode.

e Ethylenediamine cellulose immobilized palladium nanoparticles modified glassy carbon electrode.

f Palladium nano particle decorated bamboo multi-walled carbon nanotubes.

g Chronoamperometry.

h Linear sweep voltammetry.

As summarized in Table 1, the Pt/SnS electrode exhibits a competitive analytical performance compared with previously reported noble-metal-based HZ sensors. Its sensitivity in the lower concentration range (1337.6 µA mM^−1^ cm^−2^) exceeds those reported for PdNPs-EDAC/GCE (403.7 µA mM^−1^ cm^−2^) and GO/CTS/Pt/GCE (104.6 µA mM^−1^ cm^−2^). Its LOD of 2.68 µM is also lower than those of several reported electrodes, including GO/CTS/Pt/GCE, Nano-Au/Ti, and Pd/BMWCNTs. Although some comparative sensors achieve lower LODs or higher sensitivity, the Pt/SnS electrode offers a favorable combination of sensitivity and an extended upper linear range reaching 3500 µM. These comparisons establish Pt/SnS as a prospective and competitive alternative for electrochemical HZ detection.

### 3.5. Stability, selectivity, reproducibility and practical applicability

#### 3.5.1. Stability

The electrochemical stability of the Pt/SnS electrode toward HZ oxidation was evaluated by continuous cyclic voltammetric cycling in 3 mM HZ dissolved in 0.1 M NaOH at a scan rate of 0.1 V s^−1^. As shown in Fig. 8A, after 50 consecutive CV cycles, the 51st cycle retained a peak current density of 1.40 mA cm^−2^, compared with an initial value of 1.52 mA cm^−2^, corresponding to a current retention of 92.1%. Moreover, the oxidation peak potential exhibited only a slight positive shift from −0.34 V to −0.33 V. The minimal loss in current response and negligible peak potential shift indicates good electrochemical stability and good resistance of the Pt/SnS electrode to surface deactivation during repeated HZ oxidation.

**Fig. 8:**
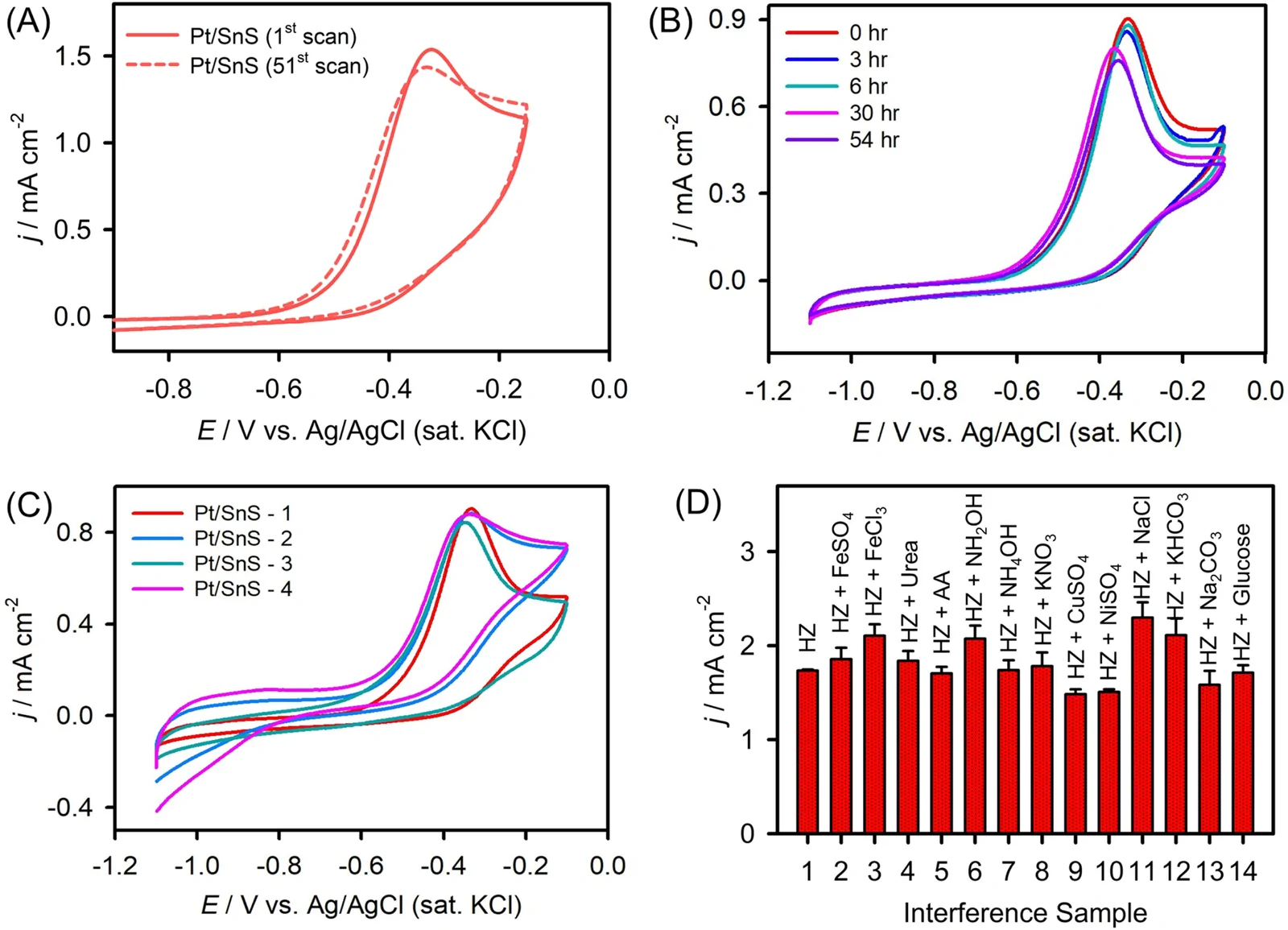
(A) CVs demonstrating the stability of the Pt/SnS electrode toward HZ oxidation, recorded in 3 mM HZ dissolved in 0.1 M NaOH at a scan rate of 0.1 V s^−1^. (B) CVs for the oxidation of 2 mM HZ recorded using a Pt/SnS electrode in 0.1 M NaOH at a scan rate of 0.05 V s^−1^ after storage for 0, 3, 6, 30, and 54 h, demonstrating the stability and repeatability of the electrode response over the storage period. (C) CVs for the oxidation of 2 mM HZ recorded using four independently prepared Pt/SnS electrodes under identical experimental conditions, demonstrating the reproducibility of the electrode fabrication and catalytic response. (D) Bar graph comparing the HZ oxidation peak current in the presence of various interfering species. The current values were extracted from the CVs shown in Fig. S4.

#### 3.5.2. Repeatability with over time and reproducibility

To further assess the stability and reproducibility of the Pt/SnS electrode, additional experiments were performed to examine the effect of storage time and electrode-to-electrode variation. For storage stability, cyclic voltammograms for 2 mM HZ oxidation were recorded using the same Pt/SnS electrode after storage periods of 0, 3, 6, 30, and 54 h under otherwise identical experimental conditions (Fig. 8A). The oxidation response remained well preserved throughout the investigated storage period (Table 2). The onset potential (*E*_onset_) and peak potential (*E*_p_) remained identical in CVs recorded in the period 0 to 6 h. The onset potential varied only slightly from −0.65 V at 0 h to −0.66 V at 54 h, while the peak potential changed from −0.34 to −0.36 V. The corresponding peak current density decreased from 0.89 to 0.75 mA cm^−2^, corresponding to approximately 84% of the initial response. These relatively small changes in the electrochemical response indicate that the Pt/SnS electrode retained its catalytic activity over the investigated storage period.

**Table 2:** Stability and repeatability of the Pt/SnS electrode over time

Storage time/hr	Onset potential, *E*_onset_/V *vs.* Ag/AgCl (sat. KCl)	Peak potential, *E*_p_/V *vs.* Ag/AgCl (sat. KCl)	Peakcurrent density, *j*_p_/mA cm^−2^
0	−0.65	−0.34	0.89
3	−0.65	−0.34	0.85
6	−0.65	−0.34	0.87
30	−0.67	−0.37	0.80
54	−0.66	−0.36	0.75

The reproducibility of the electrode fabrication was further evaluated using four independently prepared Pt/SnS electrodes under identical experimental conditions (Fig. 8C and Table 3). The independently fabricated electrodes exhibited closely comparable onset potentials ranging from −0.65 to −0.67 V and peak potentials ranging from −0.34 to −0.36 V, while the peak current densities varied between 0.83 and 0.89 mA cm^−2^. The relatively small variation in both potential and current response demonstrates good electrode-to-electrode reproducibility of the Pt/SnS fabrication procedure. Together, the storage-stability and independent-electrode measurements provide additional evidence that the observed HZ oxidation response is reproducible under the experimental conditions employed in this study.

**Table 3:** Reproducibility of four independently prepared Pt/SnS electrodes

Electrode	Onset potential, *E*_onset_/V *vs.* Ag/AgCl (sat. KCl)	Peak potential, *E*_p_/V *vs.* Ag/AgCl (sat. KCl)	Peakcurrent density, *j*_p_/mA cm^−2^
Pt/SnS – 1	−0.65	−0.34	0.89
Pt/SnS – 2	−0.65	−0.34	0.87
Pt/SnS – 3	−0.67	−0.36	0.83
Pt/SnS – 4	−0.66	−0.35	0.87

#### 3.5.3. Interference effect

The selectivity of the Pt/SnS electrode toward HZ oxidation was evaluated in the presence of 13 potential interfering species, including FeSO_4_·7H_2_O, FeCl_3_·6H_2_O, CH_4_N_2_O (urea), ascorbic acid (AA), NH_2_OH·HCl, NH_4_OH, KNO_3_, CuSO_4_·5H_2_O, NiSO_4_·6H_2_O, NaCl, KHCO_3_, Na_2_CO_3_, and glucose. Transition metal ions (Fe^2+^, Fe^3+^, Cu^2+^, and Ni^2+^) were selected because they may be present in industrial wastewater and can interact with the electrode surface or participate in competing electrochemical processes. Inorganic salts (NaCl, KNO_3_, KHCO_3_, and Na_2_CO_3_) were included to assess the influence of electrolyte composition and ionic strength on the HZ oxidation response. Nitrogen-containing compounds (urea, NH_2_OH, and NH_4_OH) were selected because of their chemical characteristics or potential coexistence in HZ-containing industrial streams, whereas glucose and ascorbic acid were included as representative organic species that may interfere with electrochemical measurements.

The bar graph in Fig. 8D, showing the HZ oxidation peak current densities extracted from the CVs in Fig. S4, illustrates the variation in the electrochemical response following the addition of each interfering species at 3 mM. The current-retention values ranged from 85.48% to 132.47%, with an average of 105.51% across the 13 tested species (Table S2). However, the average response masks species-dependent effects. FeCl_3_·6H_2_O, NH_2_OH·HCl, NaCl, and KHCO_3_ produced positive current deviations of 21.37%, 19.53%, 32.47%, and 21.74%, respectively, whereas CuSO_4_·5H_2_O and NiSO_4_·6H_2_O decreased the current response by 14.52% and 13.11%, respectively. For a quantitative assessment, a current-retention interval of 90–110% was adopted as a study-specific screening criterion, corresponding to a deviation of ±10% from the HZ-only response. Seven of the 13 tested species fell within this interval, while the remaining six exceeded it, indicating that the influence of individual interferents cannot be considered negligible in all cases.

As shown in Fig. S4, the HZ oxidation peak potential remained nearly unchanged in the presence of the tested species, and no additional redox peaks were observed within the investigated potential range. Nevertheless, the observed changes in peak current demonstrate that interference may occur even in the absence of additional redox peaks. It should also be noted that these experiments were conducted using CV with 3 mM HZ and 3 mM of each interfering species.

#### 3.5.4. Real sample analysis

To assess the practical applicability of the Pt/SnS electrode for HZ determination in complex matrices, recovery experiments were performed using simulated dye and pharmaceutical industry samples spiked with 3 mM HZ. The analytical results are summarized in Table 4. The Pt/SnS electrode exhibited satisfactory analytical performance in the investigated real samples, with recoveries ranging from 81.00% to 99.33%. The dye industry samples showed good recoveries of 97.33% for sample-1 (predominantly containing Alizarin red) and 99.33% for sample-2 (predominantly containing methylene blue), indicating that these dye constituents exerted minimal interference on the electrochemical oxidation of HZ. Similarly, the pharmaceutical sample-2 (predominantly containing fexofenadine hydrochloride) exhibited a high recovery of 96.66%, confirming the reliability of the proposed sensor in pharmaceutical matrices. In contrast, the sample-1 (predominantly containing acetaminophen) showed a comparatively lower recovery (81.00%), suggesting that certain excipients or active pharmaceutical ingredients may partially suppress the HZ oxidation current through matrix effects or competitive adsorption on the electrode surface. Nevertheless, the sensor maintained a distinct HZ oxidation response, enabling successful quantification even in this comparatively challenging matrix.

**Table 4:** Quantitative analysis of HZ in simulated real samples using the Pt/SnS electrode

Water sample	Added conc. (mM)	Obtained conc.^a^ (mM)	RSD^b^ (%)	Recovery^c^ (%)
Dye industry-1	3.00	2.92 ± 0.00	0.05	97.33
Dye industry-2	3.00	2.98 ± 0.01	0.29	99.33
Pharma industry-1	3.00	2.43 ± 0.01	0.23	81.00
Pharma industry-2	3.00	2.90 ± 0.00	0.08	96.66

a Average concentration obtained from three independent measurements using the Pt/SnS electrode.

b Relative standard deviation (RSD) of three independent measurements.

c Recovery (%) = (obtained concentration/added concentration) × 100.

The precision of the proposed sensing platform was further demonstrated by the low relative standard deviation (RSD) values, which ranged from 0.05% to 0.29%, indicating good repeatability and high measurement precision. The combination of high recoveries and low RSD values confirms that the Pt/SnS electrode possesses good accuracy, precision, and robustness for HZ determination in complex industrial and pharmaceutical samples, highlighting its potential for practical environmental and industrial monitoring applications.

## 4. Conclusion

A Pt/SnS-modified platinum electrode was successfully fabricated by immobilizing hydrothermally synthesized SnS particles onto a Pt substrate using a thin PTFE binder layer. Structural characterization by SEM, EDX, Raman spectroscopy, PXRD, and XPS provided evidence supporting the formation of the SnS-containing material. Electrochemical studies demonstrated that SnS modification enhanced the HZ oxidation response, producing more than a twofold increase in oxidation current relative to bare Pt while maintaining good operational stability. Kinetic investigations using cyclic voltammetry, chronoamperometry, and convolution potential sweep voltammetry indicated that HZ oxidation proceeds through an irreversible multistep pathway involving an overall four-electron transfer to form nitrogen, with an apparent transfer coefficient (*β*) of approximately 0.30–0.32. This behavior suggests a kinetically slow initial electron-transfer step followed by more rapid oxidation of intermediate species. The enhanced catalytic response is attributed primarily to favorable HZ adsorption and activation at the Pt/SnS interface. The electrode enabled HZ determination over two linear concentration ranges of 5–35 µM and 50–3500 µM, with a limit of detection of 2.68 µM and a limit of quantification of 8.13 µM. Interference and recovery experiments using simulated industrial samples provided an initial assessment of analytical applicability, although the variation in responses indicates that further matrix-specific validation is required. Future development could explore miniaturization through smaller-area or thin-film Pt electrodes, screen-printed electrode designs, and optimized SnS loading to reduce noble-metal consumption. Integration with a portable potentiostat and compact electronic readout could facilitate on-site hydrazine monitoring, although these approaches require further investigation and experimental validation. Overall, this study provides mechanistic insight into HZ electrooxidation at the Pt/SnS interface and establishes a foundation for the further development of practical electrochemical hydrazine sensors.

## Conflicts of interest

The authors declare no competing financial interest.

## Supplementary Material

RA-OLF-D6RA06914B-s001

## Data Availability

All data supporting the findings of this study are included in the article and in the supporting information (SI). Supplementary information: XPS analysis of the O 1s region after hydrazine oxidation (Fig. S1), XRD characterization and Le Bail refinement of the synthesized SnS catalyst (Fig. S2), cyclic voltammograms comparing SnS-modified Au, GC, and Pt electrodes (Fig. S3), EIS fitting parameters for bare Pt and Pt/SnS electrodes (Table S1), and current retention data for interfering species during hydrazine oxidation (Table S2). Additional data are available from the corresponding author upon reasonable request. See DOI: https://doi.org/10.1039/d6ra06914b.
