# Supplementary material for: SnS-modified platinum electrode for electrocatalytic hydrazine oxidation: mechanistic insights and electrochemical sensing
Source: RSC Adv. 2026 Nov 6. Online ahead of print. doi: 10.1039/d6ra06914b (PMC13741482; doi:10.1039/d6ra06914b)
Supplement: Supplementary file 1 [file RA-OLF-D6RA06914B-s001.pdf]

Supplementary Information (SI) for RSC Advances.
This journal is © The Royal Society of Chemistry

Supporting Information

SnS-Modified Platinum Electrode for Electrocatalytic Hydrazine Oxidation: Mechanistic Insights and Electrochemical Sensing

Mohammad Imran Hossain [a], S. M. Maksudur Rahman Shanto [a], Md. Tarikul Islam [a], Jahir Ahmed [b,c], M. Faisal [b,c], Jari S. Algethami [b,c], Farid A. Harraz [b,c,*], Md Mahmudul Hasan [d], Md Musfiqur Rahman [e], Mohammad A. Hasnat [a,*]

[a] *Electrochemistry & Catalysis Research Laboratory (ECRL), Department of Chemistry, School of Physical Sciences, Shahjalal University of Science and Technology, Sylhet-3114, Bangladesh (MIH: imran-che@sust.edu; SMMRS: shanto7818@gmail.com; MTI: tarikulislam038@gmail.com; MAH: mah-che@sust.edu)*

[b] *Advanced Materials and Nano-Research Centre (AMNRC), Najran University, Najran, 11001, Saudi Arabia (JA: ursjahir@gmail.com; MF: mdfaisalahsan@gmail.com; JSA: jsalgethami@nu.edu.sa; FAH: faharraz@nu.edu.sa)*

[c] *Department of Chemistry, Faculty of Science and Arts, Najran University, Najran, 11001, Saudi Arabia*

[d] *Research Organization for Nano and Life Innovation, Waseda University, Tokyo, 162-0041, Japan (MMH: hasan@aoni.waseda.jp)*

[e] *Department of Mechanical Engineering, Iowa State University, Ames, Iowa 50011, USA (MMR: md.musfiqur.rahman@maine.edu)*

***Corresponding author**: mah-che@sust.edu (Mohammad A. Hasnat), faharraz@nu.edu.sa (Farid A. Harraz)

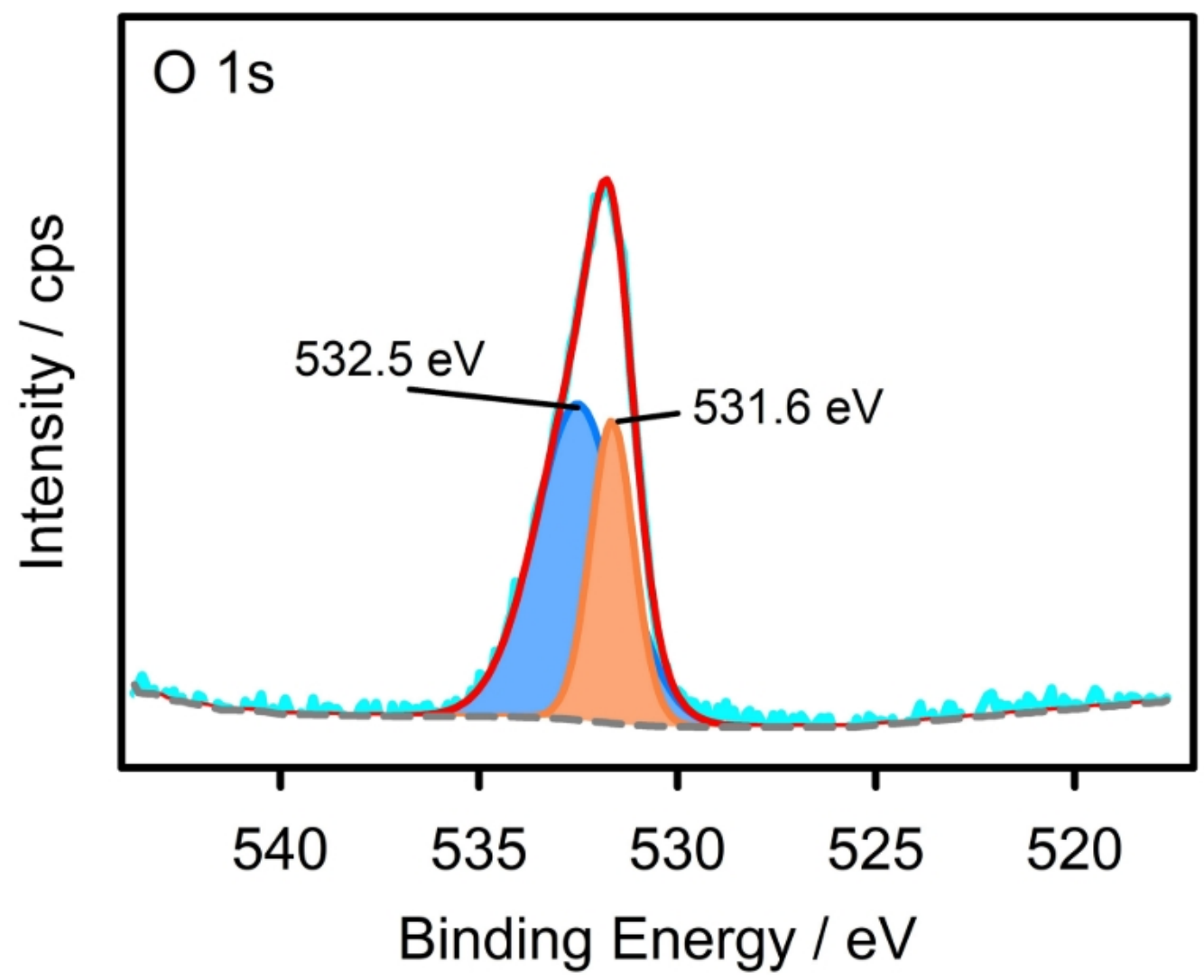

Figure S1. X-ray photoelectron spectrum (XPS) of O1s region of the SnS electrocatalyst after the HZ oxidation experiment.

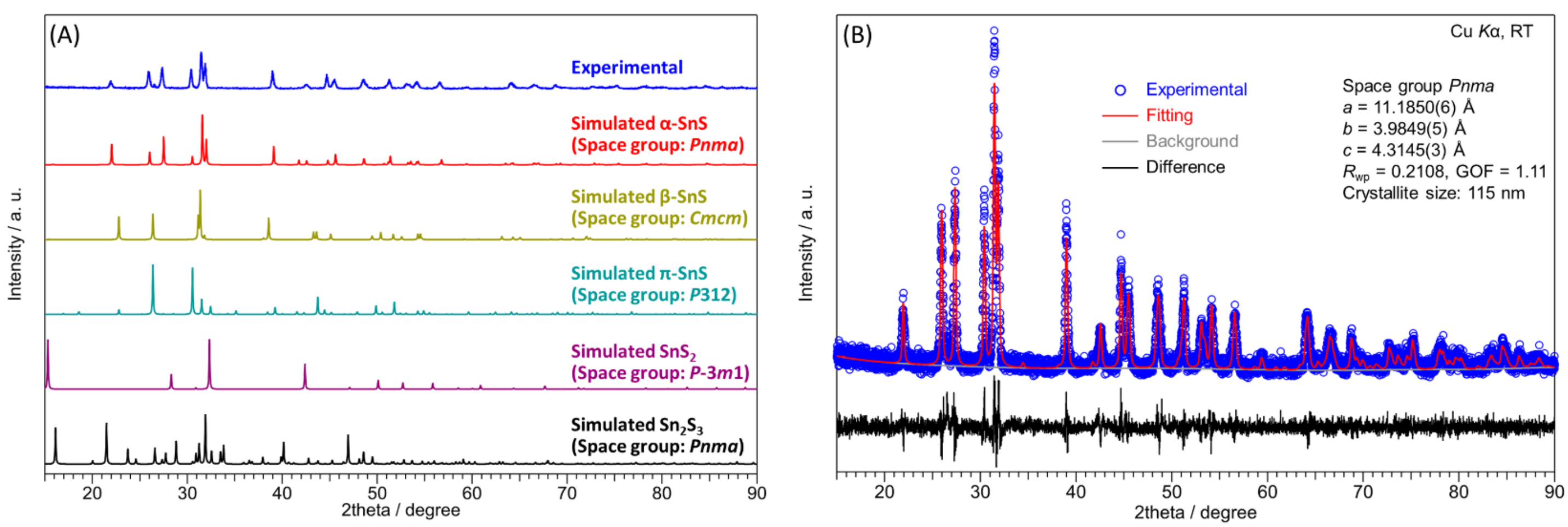

Figure S2. XRD characterization of the synthesized SnS. **(A)** Experimental pattern compared with simulated patterns of α-SnS, β-SnS, π-SnS, SnS_2, and Sn_2S_3. **(B)** Le Bail refinement of α-SnS (*Pnma*) using Cu *K*α radiation. Reproduced with permission from Ref. [1] . Copyright (2024) American Chemical Society.

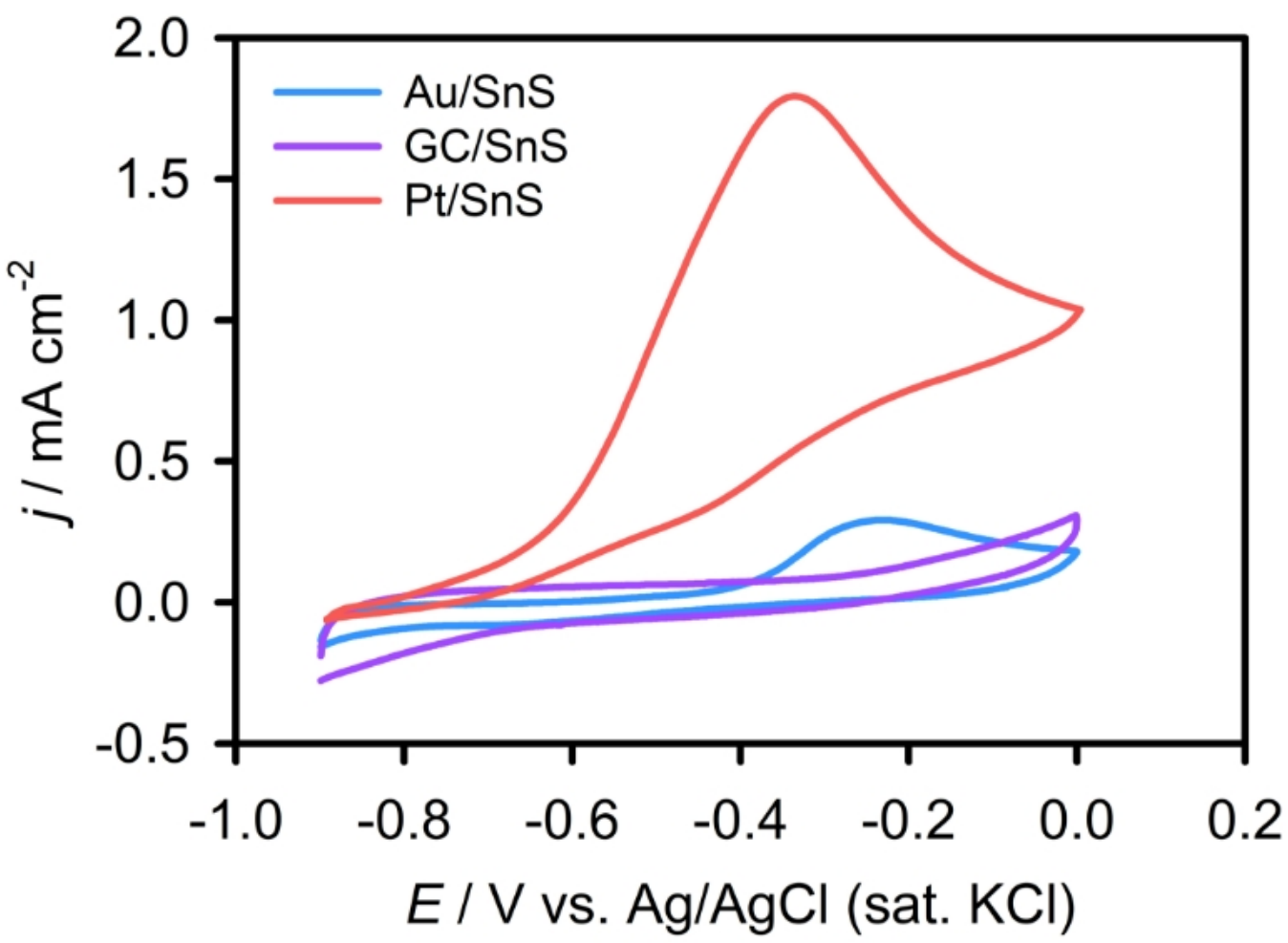

Figure S3. Cyclic voltammograms (CVs) of 3 mM hydrazine (HZ) oxidation reaction recorded in 0.1 M NaOH medium at a scan rate of 0.05 V s^{-1} using SnS-modified gold (Au) electrode, SnS-modified glassy carbon (GC) electrode, and SnS-modified Pt electrode.

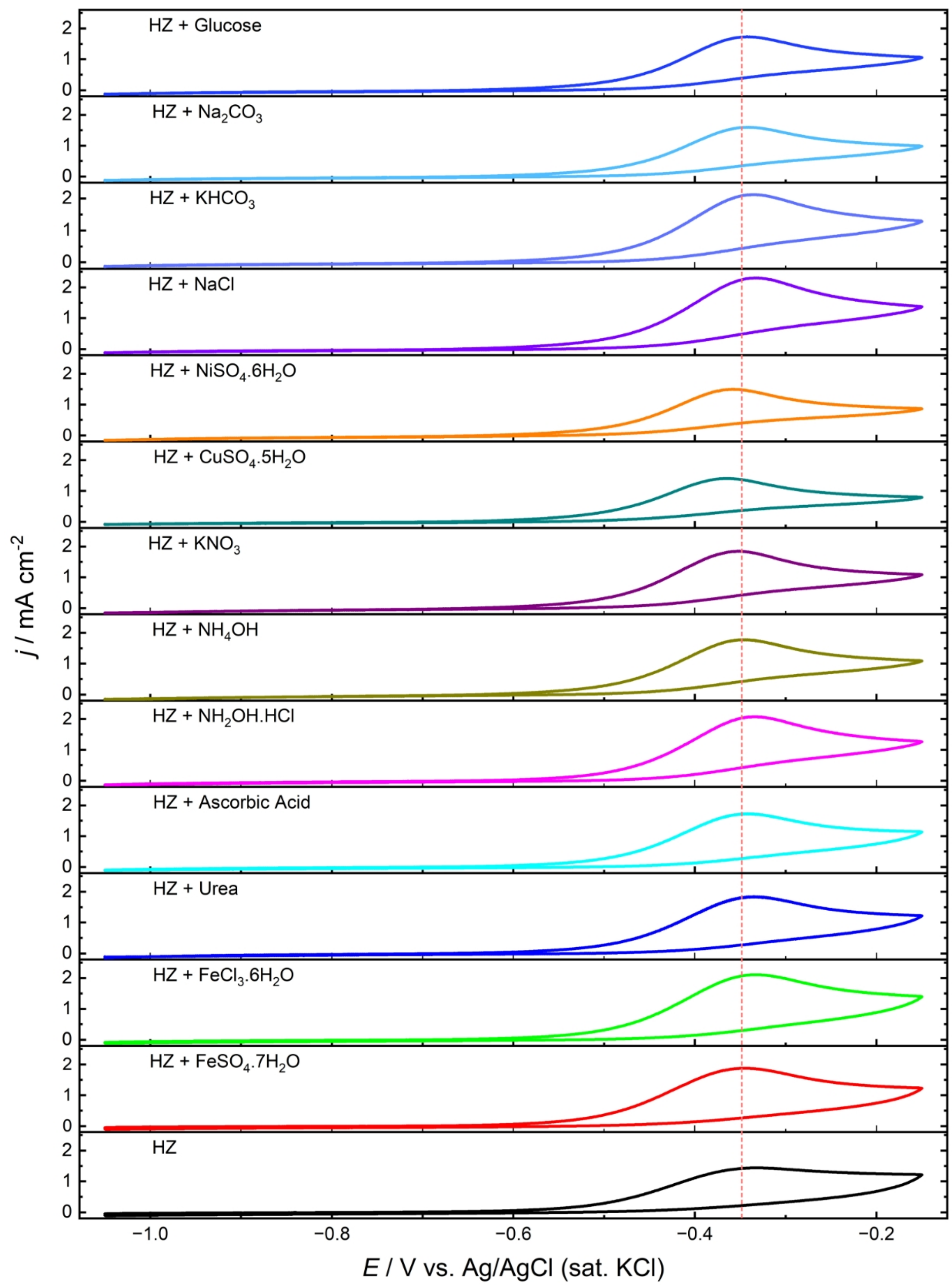

Figure S4. Cyclic voltammograms (CVs) recorded to evaluate the effect of interfering species on the electrochemical oxidation of HZ. The measurements were performed using the Pt/SnS electrode in

0.1 M NaOH containing equimolar concentrations of HZ and the respective interfering species (3 mM each) at a scan rate of 0.05 V s^{-1}.

Table S1. EIS fitting parameters for Pt and Pt/SnS electrodes.

Source Circuit	Element	Parameter	Value	Estimated Error (%)
	R_s	R	116.42 Ω	0.812
	R_p	R	20760 Ω	4.400
	CPE	Yo	6.5505×10^{-6}	2.658
Bare Pt		N	0.8482	0.491
	W	Yo	3.3687×10^{-5} μMho.s$^{1/2}$	3.285
		χ^2	0.063293	
	R_s	R	123.68 Ω	
	R_p	R	19889 Ω	5.119
	CPE_1	Yo	2.1838×10^{-6} μMho.s^N	3.327
		N	0.94756	1.107
Pt/SnS	R_1	R	15550 Ω	7.807
	CPE_2	Yo	4.7539×10^{-5} μMho.s^N	19.889
		N	0.73806	2.487
		χ^2	0.063193	

Table S2. The current retention of interfering species during HZ oxidation using Pt/SnS.

Interfering species	Current retention (%)	Relative deviation (%)

$FeSO_4.7H_2O$	106.98	+6.98
$FeCl_3.6H_2O$	121.37	+21.37
CH_4N_2O (urea)	105.97	+5.97
Ascorbic acid (AA)	98.26	−1.74
$NH_2OH.HCl$	119.53	+19.53
NH_4OH	100.29	+0.29
KNO_3	102.65	+2.65
$CuSO_4.5H_2O$	85.48	−14.52
$NiSO_4.6H_2O$	86.89	−13.11
NaCl	132.47	+32.47
$KHCO_3$	121.74	+21.74
Na_2CO_3	91.24	–8.76
Glucose	98.74	−1.26

References

1 M. T. Islam, M. I. Hossain, K. Aoki, Y. Nagao, M. M. Hasan, M. Rahaman, A. Aldalbahi and M. A. Hasnat, *ACS Appl. Energy Mater.*, 2024, **7**, 3125–3136.
